# Refractory autoimmune hemolytic anemia in a systemic lupus erythematosus patient: A clinical case report

**DOI:** 10.1002/ccr3.5583

**Published:** 2022-03-18

**Authors:** Lake R. Crawford, Natalia Neparidze

**Affiliations:** ^1^ 12228 Yale School of Medicine New Haven Connecticut USA; ^2^ Yale University School of Medicine Smilow Cancer Hospital New Haven Connecticut USA

**Keywords:** autoimmune hemolytic anemia, proteasome inhibitor, systemic lupus erythematosus

## Abstract

Warm autoimmune hemolytic anemia (AIHA) is a hematologic disorder with an incidence of 1–3 per 10^5^ individuals/year. Patients with systemic lupus erythematosus (SLE) develop AIHA in 3% of adult cases and 14% of pediatric cases. We report a case of AIHA refractory to multiple lines of treatment in a patient with SLE, who eventually responded to a proteasome inhibitor‐based combination. A patient with systemic lupus erythematous was diagnosed with symptomatic autoimmune hemolytic anemia. The patient was refractory to multiple lines of treatment including prednisone, intravenous immune globulin, methylprednisolone, rituximab, cyclophosphamide, mycophenolate mofetil, and splenectomy. She eventually had a beneficial response to a proteasome inhibitor‐based combination with bortezomib plus mycophenolate mofetil. The treatment of refractory autoimmune hemolytic anemia can be challenging. Patients with AIHA refractory to primary or secondary treatments must resort to receiving novel therapeutic modalities including combinations targeting plasma cell, T‐ and B‐cell proliferation.

## Graphical abstract

The treatment of autoimmune hemolytic anemia continues to perplex hematologists. Patients who are refractory to primary or secondary treatments must resort to receiving novel treatment modalities that do not fall within the standard of care.

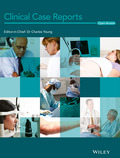

## 1 INTRODUCTION

Warm autoimmune hemolytic anemia (AIHA) is a hematologic disorder with an incidence of 1–3 per 10^5^ individuals/year and an accompanied prevalence of 17:100,000.^1^ Its pathophysiologic process involves IgG antibodies (warm agglutinins) targeting antigens on red blood cells (RBCs). This, in turn, initiates premature erythrocyte destruction through the reticuloendothelial or complement systems within the liver and spleen.^2^, ^3^ Erythrocytes coated by IgG antibodies are recognized by macrophages in the spleen and undergo membrane removal or phagocytosis.^3^ Approximately 40%–50% of cases of warm AIHA stem from an idiopathic cause—primarily from immune system activation, deficiency, or dysregulation. The remaining cases are associated with autoimmune or lymphoproliferative diseases, immunodeficiencies, infections, pregnancy, solid tumors, allogenic stem cell transplant, and drug reactions.^3^, ^4^ Furthermore, patients with systemic lupus erythematosus (SLE) develop AIHA in 3% of adult cases and 14% of pediatric cases.^5^ We report a case of AIHA refractory to multiple lines of treatment in a patient with SLE, who eventually responded to a proteasome inhibitor‐based combination with bortezomib plus mycophenolate mofetil (MMF) leading to an ongoing partial response. To our knowledge, this is the first report describing successful use of this combination regimen for a patient with heavily pre‐treated refractory AIHA.

## 2 CASE REPORT

A 44‐year‐old African American female with SLE presented to Yale New Haven Hospital as a transfer from a community hematology practice due to worsening symptomatic anemia. The patient had been diagnosed with SLE, displayed +ANA (antinuclear antibodies) titer 1:1280, +Sm/RNP (smith/ribonucleoprotein antibodies), and experienced arthralgias and fatigue; she had been evaluated by a rheumatologist and treated with hydroxychloroquine for a brief period of time. Overall, she was felt to have mildly symptomatic SLE without any active visceral organ involvement, thus SLE was not deemed active enough to merit continues systemic immunosuppressive therapy. Two years following her SLE diagnosis, she developed anemia with a hemoglobin of 7 g/dl and was diagnosed with warm AIHA with a positive direct antibody test (DAT) of 3+ IgG. At the time of diagnosis of AIHA, bone marrow biopsy demonstrated normocellular marrow with erythroid hyperplasia with no evidence of plasmacytoma, lymphoproliferative disorder, or any plasma cell neoplasm. Serum protein electrophoresis and immunofixation electrophoresis showed no monoclonal protein and CT scans did not demonstrate any hepatosplenomegaly or pathologic lymphadenopathy. She was initially treated with prednisone 1 mg/kg daily for several weeks with no response and developed worsening anemia with a hemoglobin of 4 g/dl, requiring repeated red blood cell (RBC) transfusions. Rituximab was added to the regimen without any response as hemoglobin decreased to 3.7 g/dl. The patient received RBC transfusions and was transferred to our center for inpatient management in consideration of plasmapheresis. Over the next few months, the patient received several lines of therapy including intravenous immune globulin, high dose methylprednisolone intravenously (IV) 1 g/day for 3 days, repeat doses of rituximab intravenously 375 mg/m^2^ once weekly for 4 doses in combination with steroids, five sessions of therapeutic plasma exchange, and later cyclophosphamide (1000 mg IV every 21 days for four cycles) with lack of response. Hemoglobin remained in the 3–4 g/dl range and the patient continued their transfusion requirement. Thus, further treatment was needed, and MMF at 500 mg BID was administered as the next line of immunosuppressive therapy with a transient partial response and an improvement of hemoglobin to 7.6 g/dl with subsequent worsening of anemia. The patient continued to experience episodes of lightheadedness and dyspnea on exertion.

Due to her severe refractory course, she underwent a splenectomy. The patient had a successful post‐operative recovery, but the procedure only resulted in a minimal response in hemoglobin. Post‐operatively she continued a prednisone taper in combination with MMF. Her hemoglobin remained between 6 and 7 g/dl, while still noting dizziness and requiring RBC transfusions once a week. The patient was next treated with azathioprine briefly, but this was discontinued due to lack of response and poor tolerance. Given that there was no longer a clear standard of care, a discussion held at a consensus hematology conference with multiple hematology experts in the field determined that bortezomib was the patient's best next step. The drug was commercially obtained and covered by the patient's insurance; therefore, this was not an investigational drug use and no IRB approval was needed. The patient was started on bortezomib as the next line of therapy at a dose of 1.3 mg/m^2^ subcutaneously weekly on days 1, 8, 15, and 22 of every 28‐day cycle. Within 2–3 months of therapy, she experienced a gradual hematologic response with decreasing transfusion requirements. Eventually, she achieved a partial hematologic response with hemoglobin in the 8–9 g/dl range and became transfusion independent. Given her encouraging response to bortezomib, this therapy was continued with an ongoing partial response for 24 cycles and subsequently discontinued. During this time, she had clinical improvement with decreased episodes of shortness of breath and dyspnea on exertion and remained transfusion independent. Of note was the development of transfusion hemosiderosis (peak ferritin 4770) which required iron chelation with deferasirox.

After initial course of bortezomib, the patient remained off systemic therapy with ongoing partial response for 2 years. Subsequently, she presented with increasing exertional dyspnea and developed recurrence of AIHA with hemoglobin decreasing from 9 to 7 g/dl. Once hemoglobin dropped to the 6–7 g/dl range treatment was resumed with weekly bortezomib at 1.3 mg/m^2^ on days 1, 8, 15, and 22, every 28‐day cycle. Initially, she had minimal response to bortezomib and had to resume RBC transfusions. A combination of bortezomib and rituximab was attempted; however, the patient developed a severe infusion‐related reaction during rituximab, thus infusion was interrupted. In the following days, she displayed clinical manifestations of serum sickness (fever, malaise, arthralgia, myalgia, abdominal pain, dyspepsia, and faint morbilliform rash) attributed to rituximab; therefore, the latter was discontinued. Based on previously published literature,^6^, ^7^, ^8^ consideration was given to daratumumab as the next line of the treatment; however, daratumumab administration was not feasible due to medication coverage issues. Thus, the patient received the regimen of bortezomib at a dose of 1.3 mg/m^2^ once weekly on days 1, 8, 15, and 22 of each 28‐day cycles in combination with MMF and continued this combination for six months. This combination therapy eventually resulted in partial response with an improvement of hemoglobin to 9 g/dl and transfusion independence. She continued this combination regimen, completed six cycles of bortezomib, and remained in partial response on a steady dose of MMF for 12 months. Of note, during the treatment, the patient developed a segmental pulmonary embolism, a common event in the context of AIHA, and was treated with Apixaban. Her transfusion‐related hemosiderosis was effectively treated with iron chelation therapy using oral deferasirox.

Our report is unique as it describes a case of severe AIHA completely refractory to multiple lines of treatment, later successfully managed with a proteasome inhibitor‐based combination with bortezomib plus MMF leading to a long‐lasting partial response. To our knowledge, this is the first report describing successful use of this combination regimen for heavily pre‐treated refractory AIHA.

## 3 DISCUSSION

The management and prognosis of refractory AIHA continue to perplex hematologists. This stems from science's incomplete understanding of the disease process, the complex nature of its pathophysiology, patient heterogeneity, the lack of large‐scale clinical trial data and treatment standardization. Historically, the first‐line therapy for AIHA involved the implementation of a corticosteroid regimen—which is successful in roughly two‐thirds of patients.^9^ However, the remaining one‐third of patients have to resort to additional lines of treatment. These include the following: splenectomy, rituximab, azathioprine, cyclophosphamide, and cyclosporine.^9^, ^10^, ^11^ For many years, the preferred second‐line treatment for these patients was splenectomy, but recently guidelines have begun to favor rituximab given its increased effectiveness and tolerability.^10^ Additionally, these patients suffer from multitudes of other problems stemming from their medications and physiologic states. It is well documented that patients often have infectious complications due to the immunosuppressive nature of their treatments; additionally, those that undergo splenectomy are further susceptible to serious infections.^12^, ^13^ Clinician's attention toward infectious complications through vigilant vaccinations and antimicrobial prophylaxis is crucial. Additionally, (as observed with our patient) those with AIHA often experience venous thromboembolic events.^14^ Specifically, it is estimated that 15%–33% of adults with warm AIHA will have a venous thromboembolic event.

As discussed previously, our patient was refractory to several lines of treatment. However, she attained a response to bortezomib leading to a durable partial remission. After relapse, she received a combination of bortezomib and MMF resulting in a durable partial hematologic response, transfusion independence, and significant clinical improvement. Recently, bortezomib (a 26S proteasome inhibitor)^15^ has been used as a successful therapy for immune hemolytic anemia. Bortezomib functions to inhibit the ubiquitin‐proteasome pathway which then brings about apoptosis through an augmented unfolded protein response in antibody‐producing cells.^16^, ^17^ Additionally, it has widespread immune system effects by downregulating NF‐kB’s inflammatory signaling, impairing antigenic presentation, and depleting autoreactive T cells, B cells, and plasma cells—thereby reducing antibody and autoantibody responses. Since AIHA is an immune autoantibody‐mediated process, agents suppressing B‐cell and plasma cell autoimmunity have been proven effective in its therapy. Plasma cell‐directed therapy in combination with other immunosuppressants like MMF used in our case appears effective and promising. In one case report, the authors (after previously using bortezomib to treat a patient with pure red‐cell aplasia stemming from an ABO‐mismatched stem cell transplantation) employed the same treatment in a patient with Cold Agglutinin Disease (CAD) secondary to IgM κ monoclonal gammopathy.^18^ These findings support the use of bortezomib for treatment of AIHA by targeting plasma cells responsible for producing pathogenic autoantibodies.^18^, ^19^ Daratumumab is an IgG kappa anti‐CD38 monoclonal antibody approved for treatment of plasma cell neoplasms. In a case report, a patient with AIHA following an allogeneic hematopoietic stem cell transplantation was successfully treated with improvement of hemolysis with administration of daratumumab.^20^ The role of complement is increasingly recognized in etiopathogenesis of hemolytic anemias. Thus, eculizumab is emerging as a novel treatment strategy for AIHA. In one trial, inhibition of terminal complement activation by eculizumab led to a significant reduction in hemolysis—decreasing anemia, fatigue, and the need for blood transfusions.^21^ Additionally, the recent DECADE trial found that eculizumab was able to suppressed hemolysis resulting from cold agglutinin in patients; however, circulatory symptoms of the study subjects were not significantly improved. These findings show promise for eculizumab's use in treatment for hemolytic anemia. As evidenced by this report, there are numerous different modalities that can be successfully utilized to treat AIHA, including several combination therapies. In our experience, a combination of plasma cell‐directed therapy with proteasome inhibitor along with suppression of T cells with a selective inhibitor of inosine monophosphate dehydrogenase (IMPDH) MMF proved to be an efficacious therapy. Despite multiple treatment options, many patients with AIHA remain refractory to treatment. Ongoing studies are exploring novel therapies targeting plasma cell, B‐cell lineage, and other pathways, for instance, ongoing clinical trials evaluating daratumumab hyaluronidase NCT05004259, isatuximab NCT04661033, BTK inhibitors, and other agents in adults with warm AIHA to advance current treatment. Future novel combination therapies hold promise. Further research and clinical trials are needed to achieve progress in therapy for patients with refractory autoimmune hemolytic anemia.

## CONFLICT OF INTEREST

The authors have no conflicts of interest to disclose.

## AUTHOR CONTRIBUTIONS

NN is a practicing academic hematologist in charge of the patient and given approval of the final version of the paper. LRC is a medical student in charge of conception, design, and drafting of the case report.

## CONSENT

Written informed consent was obtained from the patient to publish this report in accordance with the journal's patient consent policy.

## Data Availability

The data that support the findings of this study are available from the corresponding author upon reasonable request.
